# Organosulfur Compounds: A Review of Their Anti-inflammatory Effects in Human Health

**DOI:** 10.3389/fnut.2020.00064

**Published:** 2020-06-02

**Authors:** Ruheea Taskin Ruhee, Llion Arwyn Roberts, Sihui Ma, Katsuhiko Suzuki

**Affiliations:** ^1^Graduate School of Sport Sciences, Waseda University, Tokorozawa, Japan; ^2^School of Allied Health Sciences, Griffith University, Gold Coast, QLD, Australia; ^3^School of Human Movement and Nutrition Sciences, University of Queensland, Brisbane, QLD, Australia; ^4^Faculty of Sport Sciences, Waseda University, Tokorozawa, Japan

**Keywords:** organosulfur compounds, inflammation, chronic diseases, cytokines, NF-κB

## Abstract

Phytonutrients are widely recognized for providing protective human health benefits. Among the phytonutrients, epidemiological and experimental studies show that dietary organosulfur compounds (OSC) play a significant role in preventing various human pathological progressions, including chronic inflammation, by decreasing inflammatory mediators such as nitric oxide (NO), prostaglandin (PG)E_2_, interleukin (IL)-1β, IL-6, tumor necrosis factor (TNF)-α, and IL-17, which are all typical hallmarks of inflammation. Evidence supports OSC in reducing the expression of these markers, thereby attenuating chronic inflammatory processes. Nuclear factor-kappa B (NF-κB) is a key regulating factor during inflammation, and novel evidence shows that OSC downregulates this transcriptional factor, thus contributing to the anti-inflammatory response. *In vitro* and *in vivo* studies show that inflammation is mechanistically linked with acute and chronic pathological conditions including cancer, diabetes, obesity, neural dysfunction, etc. Furthermore, a considerable number of experiments have demonstrated that the anti-inflammatory properties of OSC occur in a dose-dependent manner. These experiments also highlight indirect mechanisms as well as potent co-functions for protective roles as antioxidants, and in providing chemoprotection and neuroprotection. In this brief review, we provided an overview of the anti-inflammatory effects of OSC and elucidated probable mechanisms that are associated with inflammation and chronic disorders.

## Introduction

**Figure 1:**
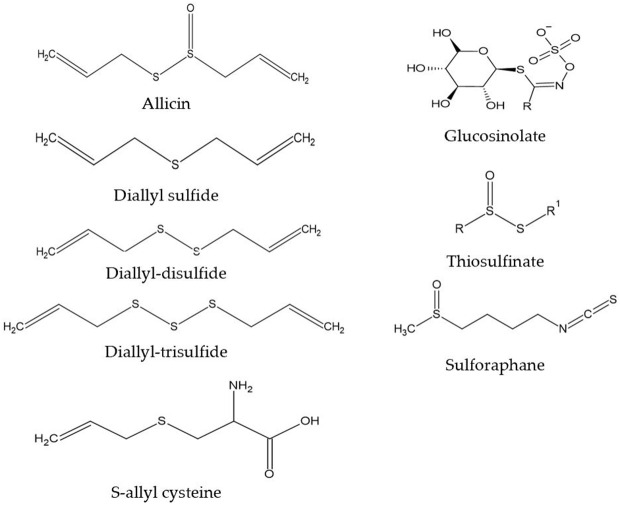
Generic structure of some organosulfur compounds.

We are obtaining a growing understanding of how bioactive compounds in plants are helpful agents to protect human health. These compounds are also referred to as nutraceuticals, or phytonutrients. These compounds are usually present in cereals (grains), pulses (legumes), vegetables, fruits, and other plant foods. Having a diet rich in plant foods, therefore, ensures the consumption of millions of phytonutrients, and provides health-protective benefits. Regularly consuming fruits and vegetables is linked with reducing the risk of chronic diseases including cardiovascular diseases (1), obesity (2), hypertension, type 2 diabetes mellitus, stroke, cancer, and chronic inflammatory bowel diseases (IBD), etc., (3). This risk reduction is purported to be from fruits, vegetables, whole grains, and other plant-based foods containing significant amounts of phytonutrients that exert potential attributes beyond those obtained from basic nutrition. Dietary phytonutrients are very diverse, and are as such classified as phenolics, alkaloids, nitrogen-containing compounds, organosulfur compounds (OSCs), phytosterols, and carotenoids (4). Each class is divided into further classes, but herein within the scope of this review, only the OSC group will be discussed. The OSC group includes isothiocyanates, indoles, allylic sulfur compounds, and sulforaphane (SFN) (5). Figure 1 listed some basic generic structure of some organosulfur compounds. These compounds are widely known for their unique medicinal properties and health benefits, i.e., they act as antioxidants to scavenge free radicals (6), and possess antimicrobial and anti-inflammatory properties (7). Numerous health benefits of these compounds are evidenced against chronic diseases by e.g., their cardioprotective effects of reducing low-density lipoproteins (LDL), and anti-carcinogenic effects by detoxifying carcinogens or toxicants (8). Vegetables in the Allium and Brassica (Cruciferous) genus, i.e., onion, garlic, broccoli, cabbage, cauliflower, etc., are good sources of OSCs. These are widely consumed by the general population, with well-documented benefits (9, 10).

Vegetables of the Allium genus can be easily identified by their typical pungent flavor and aroma, due to the presence of oil-soluble or volatile sulfur compounds (diallyl sulfides) (11). However, some less odorous OSCs, i.e., S-allyl cysteine (SAC) or S-allyl mercapto cysteine (SAMC) found in aqueous extracts of garlic are present in vegetables of the Allium genus and are water-soluble in nature (12). When garlic is cut or crushed into pieces, OSC is naturally activated within a short period of time (4). Also, crushed raw garlic contains high concentrations of allicin, and it is allicin that is responsible for most of the potential pharmacological activities (13). The strong flavor of onion is identified due to some intermediary sulfur compounds, namely thiosulfinates, thiosulfonates, and mono-, di-, and tri-sulfides (14). Glucosinolates (GST) are natural plant compounds in Brassica (Cruciferous) genus, also known as precursors of isothiocyanates. While cellular disruption by the enzyme myrosinase is caused by chewing, food preparation or damage by insects causes the hydrolysis of GST into several bioactive compounds i.e., isothiocyanates, thiocyanates, and others (15). SFN is an important dietary isothiocyanate that is a hydrolytic product from glucoraphanin (4-methyl-sulfinyl butyl glucosinolate). A high concentration of glucoraphanin is available in broccoli, around 0.8 to 21.7 μmol/g of dry weight (DW) (16). In broccoli sprouts, the SFN concentration (1,153 mg/100 g DW) is about ten times greater than mature broccoli (44–171 mg/100 g DW) (17). SFN is an indirect antioxidant, that induces immunomodulatory, anti-inflammatory, anti-microbial, cardioprotective, chemopreventive, and neuroprotective effects (18–23).

Inflammation is a physiological state that occurs when our body is exposed to harmful endogenous or exogenous materials, injury or trauma (24). It is mainly a protective response, that is essential for survival. The regular inflammatory response involves the accumulation of white blood cells, especially neutrophils, macrophages, and plasma proteins in sites of injury or foreign particle location, to selectively eliminate cells and to restore homeostasis (24, 25). Consequently, secondary responses are also often visible such as redness, pain, fever, and swelling (24). Such an inflammatory response is called acute inflammation, but if it lasts for prolonged periods, it is then termed chronic, or homeostatic inflammation. Further, activating the innate immune system can also induce a more chronic inflammatory response (26). Therefore, regulating both metabolic and immune responses is essential to maintain central homeostasis, because a deviation may occur in normal immune responses if there is any chronic disturbance in metabolic homeostasis by endogenous or exogenous inducers (27). This is followed by a disrupted metabolic function that results in over- or undernutrition (28). This kind of metabolically triggered chronic inflammation is termed *metaflammation*, also known as *low-grade inflammation*, that is associated with many pathophysiologies (28), including obesity, type 2 diabetes and cardiovascular diseases (28, 29). Therefore, attention should be paid to OSC as they exert physiological effects against both acute and chronic inflammation, not only *in vitro*, but also in humans (30). Subsequently in this review, we will describe the principles of anti-inflammatory regulation by OSC, and the potential for OSC to contribute to alleviating common metabolic disorders.

## Effects of OSC in Preventing Acute and Chronic Inflammation

### Average Consumption and Bioavailability of OSC

Ensuring good nutrition is essential to live a disease-free healthy life. According to a survey conducted by the Japanese government in 2011–13, cruciferous vegetable consumption topped a list among all commonly consumed vegetables, made up of around 21% of purchased volume for cabbage, onion, and broccoli (31). However, other statistics from a population-based cohort study in Spain revealed that the average intake of cruciferous vegetables among Spanish people was 11.3 g/day, the lowest consumption compared with other European countries (32). Some studies propose that the bioavailability and metabolic rate of oil-soluble OSC in garlic, like allicin and other allicin-derived compounds, is rapid, and absorbed intestinally; however, the administration of a high dose (60 mg) of pure allicin, or a lower dose of 25 g from crushed garlic did not appear in human blood, urine or feces within 48 h of consumption (33). On the other hand, SAC is a stable water-soluble OSC in garlic, that can be absorbed easily in the gastrointestinal tract within 30–60 min and detected in liver, kidney, and plasma after oral intake in animals (34). In urine it was identified as a metabolite of N-acetyl-SAC, transformed by N-acetyltransferase. The bioavailability of SAC is 98.2% in rats, 103.0% in mice and 87.2% in dogs (34). On the contrary, the bioavailability of cruciferous vegetables is measured by analyzing their metabolites and conjugated products excreted through urine. For example, the bioavailability of SFN derived from broccoli is determined by the excretion of GSH conjugates excreted through urine within 24 h post-ingestion, which indicates the transport of bioactive compounds throughout the body (35, 36). Thus, the bioavailability of OSC can differ according to the origin and dose (37).

### Inflammation Induced by Exogenous Stimuli

Inflammation is an important part of our body's immune response. In the case of acute injury, neutrophils play a crucial role in enhancing the inflammatory response by producing cytokines, whilst neutrophils are considered as both inflammatory effectors and immunoregulatory cells (38). Exogenous stimulation can be caused by mechanical, physical, chemical or biological stimulation, or a combination of these stimuli (39). Researchers in the field of sports science have revealed that exercise has both inflammatory and anti-inflammatory effects (40). In 1994, it was hypothesized that unaccustomed exercise causes muscle damage mainly by metabolic and mechanical stressors (41). In fact, an influx of inflammatory cytokines such as tumor necrosis factor (TNF)-α, interleukin (IL)-1β, IL-6, IL-8, and IL-12 are secreted due to inflammation, whilst neutrophils and macrophages also accumulate in the inflamed body part or organ (42–44). Furthermore, during exercise, the movement of electrons through the mitochondrial electron transport system results in increased levels of peroxides, superoxide radicals, their by-products, and other ROS (45). Some studies suggest that dietary immunostimulants i.e., antioxidants, polyphenols, etc., can positively improve exercise-induced changes in immune function (46–48). Therefore, evidence suggests that OSC can modulate our immune system, and are able to ensure protection against inflammation and oxidative stress (19, 49–51). A couple of experimental studies with animal models have been conducted with SFN obtained from *Brassicaceae* vegetables, to examine its protective effect against muscle inflammation. It was revealed that SFN could reduce exhaustive exercise-induced muscle damage as well as modulate the muscle redox environment by inducing transcription factor NF-E2-related factor 2 (Nrf2)-dependent phase 2 enzymes (52). Also, SFN has been shown to reduce muscle inflammation by acting as an indirect antioxidant and anti-inflammatory compounds, by activating the Nrf2-induced inhibition of nuclear factor-kappa B (NF-κB) signaling pathway (53). Regardless of whether OSC comes from Allium or Brassica genus, it seems to serve the same role against inflammation (54).

### Inflammation Induced by Endogenous Stimuli

Endogenous inducers of inflammation are responses produced by stressed, malfunctioning or dead cells, damaged tissues, plasma, or the extracellular matrix. Based on the nature and location of inflammation in our body, a common mechanism is followed by all inducers, which is summarized in the following process. Firstly, detrimental stimulation is recognized by cell surface pattern recognition receptors (PRRs); secondly, inflammatory pathways are activated; thirdly, inflammatory mediators are released; and finally, inflammatory cells are recruited (55). At the initial point of inflammation, PRRs recognize molecules of pathogens and damaged cells by orientating pathogen-associated molecular patterns (PAMPs) and/or damage-associated molecular patterns (DAMPs) from endogenous stress. Therefore, activation of inflammasomes [a group of cytosolic protein containing nucleotide-binding oligomerization domain (NOD)-containing protein 2-like receptors (NLRs)] throughout the innate immune response promotes the production of pro-inflammatory cytokines (56). Furthermore, it is necessary to ensure a balance of expression between pro- and anti-inflammatory cytokines to maintain regular cell and organ function (57). Chronic dysregulation of an increased volume of pro-inflammatory cytokines may lead to atherosclerosis, type 2 diabetes and other chronic diseases (28, 58). Thereby, high circulating inflammatory substances are released from adipose tissues at the onset of metabolic disorders, which is termed low-grade systemic inflammation (59). One of the possible mechanisms of OSC against carcinogenesis could be due to the xenobiotic mechanism of phase 1 and phase 2 enzymes (60). According to previous research, an association has been found between elevated levels of circulating inflammatory indicators such as IL-6 and TNF-α, and the development of chronic diseased conditions, e.g., type 2 diabetes, atherosclerosis, and cardiovascular diseases, or low-grade systemic inflammation (61–63). Moreover, circulating acute-phase protein, C-reactive protein (CRP), is also elevated alongside IL-6 and TNF-α (64), which further highlights the risks of developing low-grade systemic inflammation. Several investigations have been performed to identify the role of OSC against inflammation. One such study has used human embryonic kidney cell line 293 (HEK293) with sulfur fertilized garlic powder extracts (GPE, the sulfur amount rose to 190 mmol/kg), to determine lipopolysaccharide (LPS, at a final concentration 10 μg/L, 100 mg/L GPE)-induced inflammation (63). Results were compared between unfertilized and fertilized GPE and evidenced that fertilized GPE could significantly reduce NF-κB, IL-1β, and TNF-α, and LPS-induced liberation of anti-inflammatory IL-10 was not affected by fertilized GPE (65). The aging process of garlic produces odorless sulfur compounds, including the conversion of unstable allicin into a stable and more beneficial one, with a substantially increased amount of S-allyl cysteine and S-allyl mercapto cysteine. Furthermore, S-allyl compounds are higher in commercially produced aged garlic compared with fresh garlic (66). As an example, a 6-week double-blind, randomized, placebo-controlled nutritional intervention study was conducted using 51 healthy adult obese human subjects supplemented daily with aged garlic extract in capsule form, or placebo. The subjects were instructed to consume three capsules (3.6 g aged garlic extract/day) with food throughout the study timeline. Besides other biomarkers, supplementation leads to a decrease in serum IL-6 and TNF-α concentrations below those at baseline (67). This exemplifies how aged garlic supplementation might prevent the progression of obesity-induced inflammation.

### Influence of OSC on Transcriptional Regulation of Pro-inflammatory Mediators

**Figure 2:**
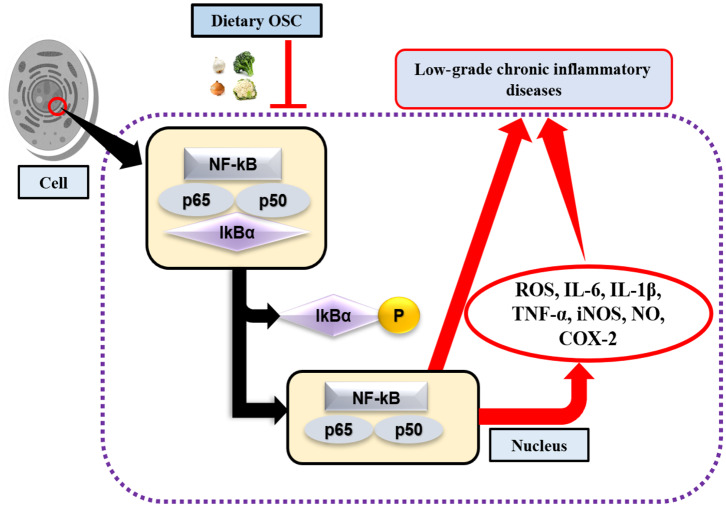
OSC in activation of NF-κB signaling pathway. Lack of dietary OSC may activate the transcription factor NF-κB through the phosphorylation of IκB complex at the site of inflammation. NF-κB along with the subunits (p65 and p50) enters into the nucleus, thereby induces production of ROS, pro-inflammatory cytokines (IL-6, IL-1β, TNF-α) and enzymes (iNOS, COX-2), while their chronic production leads to increase risk of low-grade chronic inflammatory diseases.

Toll-like receptors (TLR) are highly conserved PRRs expressed in immune cells (i.e., macrophages). They recognize inflamed areas and activate gene transcription factor NF-κB, the pivotal transcription factor with five subunits (p50, p52, p65, RelB, and cRel) that subsequently regulate more than 400 different genes, including inflammatory cytokines and pro-inflammatory enzymes cyclooxygenase (COX)-2, and 5-lipoxygenase (LOX) (68, 69). Furthermore, at the site of inflammation, the release of pro-inflammatory cytokines and the accumulation of ROS are also regulated by the transcriptional activation of NF-κB through the phosphorylation of IκB (Figure 2). Activated NF-κB with attached subunits p65 and p50 translocate into the nucleus from the cytoplasm to induce pro-inflammatory genes (70). The production of pro-inflammatory cytokines may upregulate the expression of inducible nitric oxide synthase (iNOS), which leads to the increased production of nitric oxide (NO) (71). COX-2 is another rate-limiting enzyme and is responsible for the production of the prostaglandin E_2_ (PGE_2_). Lee et al. (72) performed an experiment on garlic-derived OSC (ajoene) cultured with LPS activated RAW 264.7 macrophages. OSC suppressed NO and PGE_2_ production, and the mRNA expression of pro-inflammatory cytokines IL-1β, IL-6, and TNF-α. Additionally, LPS challenged or activated macrophages displayed repression of NF-κB (72). Another study elucidated synergistically that SFN in combination with other bioactive compounds such as phenethyl isothiocyanate and curcumin could work more effectively against inflammation by reducing iNOS and COX-2 protein expression, and NO, PGE_2_, TNF-α, and IL-1 production (73). SAC, a potential cholesterol-lowering compound present in aged garlic extract, has roles in preventing oxidized LDL (ox-LDL)-induced cell damage by reducing intracellular glutathione (GSH) depletion, and TNF-α or H_2_O_2_-induced NF-κB activation (74). Also, it was observed that elevated levels of ox-LDL are one of the major reasons for atherosclerosis (75). Allyl sulfides, isothiocyanates, and indoles are the most studied OSCs. Garlic and onion are also considered as natural hypolipidemic spices, and lower serum cholesterol levels are induced if applied in appropriate doses and durations (76–78). In addition, OSC possesses antioxidant effects by acting as an inducer of phase 2 enzymes, named as the antioxidant response element (ARE) (12). In an animal model of neurodegeneration, it was evidenced that SFN could modulate the transcription factor Nrf2-dependent phase 2 enzymes to reach the central nervous system (CNS) by crossing the blood-brain barrier (BBB), thereby showing neuroprotective effects (79). As such, to some extent, administering OSC rich foods in the diet may downregulate the expression of NF-κB, and inhibit the production of pro-inflammatory mediators.

Chronic activation of inflammasomes, as well as the production of pro-inflammatory cytokines induces inflammatory cascades, that further lead to the genesis of chronic inflammatory disease. Among the cytokines, IL-1β, and IL-18 are particularly produced actively by interacting with TLRs (80). During chronic inflammation, the production of the major pro-inflammatory enzymes, iNOS and COX-2, increase and contribute toward developing cardiovascular diseases (81). According to previous research, SFN suppresses the expression of pro-inflammatory enzymes by blocking the mitogen-activated protein kinase (MAPK) signaling cascade, thus showing preventive effects against chronic inflammation (82). This mechanism, to some extent, is supportive to protect against neurodegenerative disorders and supports anti-inflammasome properties (80). In response to pathogens, macrophages undergo a metabolic shift, and exhibit the Wargburg effect (as traditionally associated with cancer cells) (83). OCS has been shown to influence cancer cell metabolism through inhibiting the activity of histone deacetylases (HDACs) and histone acetyltransferases (HATs), thereby inducing an epigenetic mechanism to normalize cancer cell metabolism (84).

Microglia cells of the CNS also release pro-inflammatory cytokines and mediators which cause neural damage (85). SFN can protect neuro-inflammation or inflammation in the brain by attenuating such inflammatory markers, as modeled by LPS-stimulated microglial cells *in vitro* (86). A considerable number of studies have identified that inflammation is associated with oxidative stress and pathophysiological domains of depression, and there are some depression-induced neuropsychiatric disorders that can be identified by an increase in serum levels of circulating inflammatory biomarkers (87, 88). Though depression is a multifaceted disorder, inflammatory biomarkers could provide useful clinical information for initial treatment. The transcription factor Nrf2 is associated with the protection against depression or oxidative stress through anti-inflammatory mechanisms. For example, SFN was administered as an antidepressant in an LPS-challenged inflammatory model of depression (89). To examine the anti-inflammatory effects of SFN, iNOS expression was measured in the hippocampus of the mice and was shown to be significantly reduced, compared with LPS-inducing iNOS by 60% (89). To summarize, this anti-inflammatory action of SFN in conjunction with its antioxidant function could introduce a novel therapeutic treatment for diseases. Links already exist, for example, with chronic obstructive pulmonary diseases (COPD) (90), with great potentials for future examination of the links between the anti-inflammatory properties of OSC, and the progression of chronic disease prevention. However, besides the anti-inflammatory properties, the effects of SFN on inflammasomes are also being studied, with reports emerging of SFN reducing the expression of the cytokines IL-1β and NOD-like receptors (NLRP3 and NLRC4), but not for AIM2 (absent in melanoma 2) (91). Similar effects have also been observed from the sulfur extract derived from garlic and onion in inhibiting the NLRP3 inflammasomes activation (92). Therefore, OSC may not be an ideal modulator for the inactivation of inflammasomes, rather than inhibiting other inflammatory regulators.

## Experimental Evidence of OSC-Mediated Anti-Inflammatory Effects, and Their Associated Mechanisms

### Anti-inflammatory Effects of OSC on Chronically Induced Low-Grade Systemic Inflammation

For decades, research has been conducted into the regulation and/or suppression of pro-inflammatory mediators through dietary sources in the context of low-grade systemic inflammation, where a series of pro-inflammatory markers are released into the circulation. Table 1 represents a summary of some key pieces of evidence surrounding the anti-inflammatory effects of different OSCs. In an *in vitro* analysis, Heiss et al. (94) proposed that the anti-inflammatory effects of SFN could also be used as a drug for cancer chemo-prevention, through the transcriptional down-regulation of NF-κB, as well as attenuating iNOS and COX-2 expression, and the secretion of TNF-α. Lee et al. (96) demonstrated that diallyl sulfide (DAS) extracted from garlic oil, a principal flavoring compound, can reduce joint-inflammation induced by monosodium urate (MSU) crystals and IL-1β, in both *in vivo* and *in vitro* models. They also showed that the pathway of action involves downregulating COX-2 expression as well as PGE_2_, which is attributed to the attenuation of NF-κB activation. The anti-inflammatory effects of garlic sulfur compounds have also been shown to prevent atherosclerosis by inhibiting the production of NO by suppressing iNOS mRNA, as demonstrated in an LPS-stimulated macrophage cell line RAW264.7 (98). Furthermore, remarkable anti-inflammatory effects of OSC can be observed if applied in an appropriate dose and concentration. For example, compared to the OSC, DAS (1–10 μM), diallyl disulfide (DADS, 0.1–0.2 μM) and allyl methyl sulfide (AMS, 2–20 μM) derived from garlic oil against endotoxicity, induced diverse effects on both pro- and anti-inflammatory cytokines, in a concentration-dependent manner. In this study, a strong correlation was also found between DAS-induced suppression of NO, PGE_2_ and the decreased production of cytokines (102). If applied in a high concentration (~200 mg/kg body weight), an imbalance between chemical stress and response capacity leads to contradictory results along with adverse side effects, i.e., acute pulmonary edema, toxicity to the heart, brain, liver, and other organs (103). One study was performed with both low (50 mg/kg) and high (500 mg/kg) doses of garlic extract, and concluded that intraperitoneal administration of high-dose garlic extract causes damage to the lungs and liver in rats (104). Similarly, low (50 mg/kg) and high (500 mg/kg) amounts of aqueous onion extract were administered both orally and intraperitoneally, and resulted in damaging effects to the lung and liver with intraperitoneal application compared with oral application, including a 25% mortality rate in the treatment group (105).

**Table 1:** Research elucidating the *in vitro* and *in vivo* anti-inflammatory effects of organosulfur compounds (OSCs).

**Compound name, source**	**Experimental model; cell line/animal type**	**Dose**	**Key observations of inflammatory mediators**	**Proposed mechanism of anti-inflammatory effects**	**References**
DATS, Garlic	LPS induced model; RAW 264.7 macrophages	LPS 100 ng/mL DATS 0, 10, 20, 30, 40 μM	NO, and iNOS protein expression was inhibited and PIC IL-1β and TNF-α reduced the expression at the transcriptional level.	Suppressed DNA binding activity and nuclear translocation of NF-κB p65, as well as inhibiting IκB degradation.	(93)
SFN, Broccoli	LPS induced model; RAW 264.7 macrophages	LPS 500 ng/mL SFN 10 or 20 μM	Expression of TNF-α, iNOS and COX-2 protein was inhibited. Also, iNOS mRNA expression downregulated.	Inhibition of NF-κB DNA binding and of transactivation of κB-dependent genes.	(94)
SFN, Broccoli	LPS induced model; RAW 264.7 macrophages	LPS 50 ng/mL SFN 20 μM	Inhibited COX-2 protein and mRNA expression.	Modulation of core promoter elements (NF-κB, C/EBP, CREB, and AP-1) in the COX-2 transcriptional regulation.	(95)
SFN, Broccoli	LPS induced model; RAW 264.7 macrophages	LPS 1 μg/mL SFN 10 or 20 μM	Reduced PIC, iNOS and NO expression.	Activation of Nrf2/HO-1 signal transduction pathway.	(71)
DAS, Garlic	MSU crystals and IL-1β: HIG-82 synovial cell	MSU crystals 2 mg/mL, IL-1β 10 ng/mL DAS 20 mM	COX-2 and PGE_2_ gene expression were inhibited by the presence of DAS.	Prevent the activation of NF-κB.	(96)
Ajoene, Garlic	LPS induced model; RAW 264.7 macrophages	LPS 1 μg/mL Ajoene 20 μM	Inhibited production of iNOS, COX-2 at the transcriptional level, and expression of TNF-α, IL-1β, and IL-6 mRNA.	Prevent the activation of NF-κB and decreased the phosphorylation of p38 and ERK.	(72)
Allicin and ajoene, Garlic	LPS induced model; RAW 264.7 macrophages	LPS 1 μg/mL Ajoene 10 μM, Allicin 50 μM	Reduced expression of iNOS in activated macrophages.	The inhibitory effect is mediated via reduction of iNOS mRNA expression rather than the effect on the iNOS enzymatic activity.	(97)
SAC, Garlic	LPS induced model; RAW 264.7 macrophages	LPS 1 ng/mL SAC 20 μM	Inhibited production of NO by suppressing iNOS mRNA and protein expression.	Suppressed activation of NF-κB.	(98)
DAS, DADS, and AMS, Garlic	LPS induced model; RAW 264.7 macrophages	LPS 330 ng/mL DAS 1-10 μM; DADS 0.1-0.2 μM; AMS 2-20 μM	PIC TNF-α, IL-1β, IL-6, and AIC IL-10 concentration were inhibited by DAS. DADS increased the production of PIC IL-1β and IL-6 and decreased AIC IL-10 production. AMS suppressed TNF-α, NO and enhanced AIC IL-10.	Mediated by reducing inflammatory enzymes (iNOS and COX-2) activity.	(99)
GE, Garlic	LPS stimulated model; PBMCs	LPS 1 μg/mL GE 1-100 μg/mL	Monocyte cytokine and their percentage of producing TNF-α, IL-6, and IL-8 inhibited and IL-10 increased significantly.	Mediated by inhibiting Th1 and inflammatory cytokines production.	(100)
SFN, Broccoli	LPS induced model; Endothelial cell line ECV304	LPS 500 ng/mL SFN 5-20 μM	Suppressed expression of COX2 and iNOS protein.	Mediated by suppressing the phosphorylation of ERK1/2, JNK, and p38.	(82)
SFN, Broccoli	CCI induced neuropathic pain model; Male C57 BL/6J mice	SFN 0.1-100 mg/kg by i.p. injection Control vehicle: Saline	Attenuated mRNA and protein expression of COX-2, iNOS and mRNA expression of TNF-α, IL-1β, IL-6. Also elevated IL-10 mRNA expression.	Reduced inflammatory cytokines expression by inhibiting inflammatory enzymes (iNOS, COX-2) expression.	(101)
SFN, Broccoli	LPS induced ALI mice model; Male BALB/c mice	SFN 50 mg/kg by i.p. injection Control vehicle: PBS LPS 25 μg in 50 μL PBS	Decreased NO, COX-2 (PGE_2_) and NF-κB, IL-6, and TNF-αprotein expression in comparison to the control group.	Reduced ALI injury through the Nrf2/ARE pathway.	(20)
SFN, Broccoli	Dystrophin deficient *mdx* mouse model; C57BL/10ScSn-Dmd*mdx*/NJU mice	SFN 2 mg/kg by gavage Control vehicle: corn oil	Reduced mRNA expression of TNF-α, IL-1β, IL-6, NF-κB in muscle cells. Inverse correlation observed between TNF-α mRNA, and Nrf2 protein expression.	Increased expression of HO-1 and Nrf2, which decreased the expression of NF-κB, and phosphorylated IκB.	(53)

*DATS, diallyl trisulfide; PIC, pro-inflammatory cytokines; MSU, monosodium urate; SAC, S-allyl cysteine; AIC, anti-inflammatory cytokines; PBMCs, peripheral blood mononuclear cells; Th1, T-helper-1; GE, garlic extract; CCI, chronic constriction injury; i.p., intraperitoneal; PBS, phosphate buffer saline; ALI, acute lung injury*.

An increased concentration of circulating inflammatory components (in plasma and urine) has also been reported for type 2 diabetes (106). Treatment with OSC (AMS) showed a reduction in the protein expression of pro-inflammatory cytokines in streptozotocin (STZ)-induced experimental rats (107). Moreover, extracts of OSC showed significant effects in reducing the risks of cancers (108), especially prostate cancer, through suppressing inflammation and altering cell signaling pathways (109, 110). Based on our knowledge of their molecular mechanisms and biological activity, these OSC could be metabolized inside our body in multiple ways. Chronic inflammation of the digestive tract or the pathogenesis of inflammatory bowel diseases (IBD) also secrete mucosal cytokines (111). IBD occurs due to an imbalance in bacterial composition and disturbance in microbial functionality. Human microbiota releases beneficial metabolites i.e., short chain fatty acids (SCFA) notably acetate, butyrate and propionate (112). Besides that, SCFA can interact with neutrophils at the site of inflammation, and regulates production of inflammatory cytokines besides minimizing the harmful effects of ROS (113, 114). Some OSCs like isothiocyanates and garlic showed potential epigenetic activities in the modification of bacterial DNA or genes (115). Garlic as a source of prebiotics also has significant influence on creating a nutritional niche in GI tract. A study was conducted with IBD patients, examining the use of garlic extract as a therapeutic agent. Human whole blood cells and peripheral blood mononuclear cells (PBMC) were treated with various concentrations of garlic extract and showed immunosuppressive activity by significantly decreasing the amount of IFN-γ, TNF-α, and IL-2 produced by T cells (100).

### Anti-inflammatory Effects of OSC Through Multiple Signaling Pathways

DAS is a potential natural phytonutrients from garlic and may prevent oxidative stress-induced inflammation of the airway (116). For example, an *in vitro* model was performed examining DAS (7.5 μM) treatment against TNF-α (10 ng/mL) exposure. Inflammation was inhibited by DAS through the downstream regulation of NF-κB and activator protein-1 (AP-1), and the ROS-induced expression of P13K/Akt signals (116). SFN in broccoli and cabbage also exert significant roles in downregulating pro-inflammatory cytokines and up-regulating anti-inflammatory cytokines, by interfering with the NF-κB or Nrf2 pathway. To some extent, the activation of the Nrf2/ARE pathway is also associated with the reduction of inflammation through repressing p38 MAPK (117), whilst COX-2 and iNOS expression can also be manipulated by SFN through the p38 MAPK-dependent pathway (118). Moreover, some other inflammatory genes, i.e., monocyte chemoattractant protein (MCP)-1, vascular cell adhesion molecule (VCAM)-1 and TNF-α expression can also be influenced by the p38 MAPK pathway (119, 120). Recently, an interesting result was obtained from a study conducted to examine the effects of SFN administration on reducing muscle inflammation in a dystrophin-deficient mdx mouse model. Administering SFN reduced muscle inflammation by the Nrf2-induced inhibition of NF-κB (p65) mRNA, and reduced the expression of pro-inflammatory cytokines (53). To this end, we can conclude that OSC is acting as a potential anti-inflammatory compound and actively induces effects in a dose-dependent manner.

## Discussion

Phytonutrients in our diet play emerging roles in preventing inflammation and modulating metabolic pathways associated with developing chronic disorders (121). Existing data to date suggest that worldwide, heart disease, stroke, and COPD are the top three leading causes of death (122). A proper diet with a balance of phytonutrients may lower the risk of such diseases (121). Depending on their biosynthesis and biogenesis, food-derived bioactive compounds are influencing human health to prevent and/or remodel the genesis of chronic metabolic disorders. In humans, a considerable amount of IL-6 (15–35%) is also released from adipose tissue by T-cells and macrophages (25, 123), and there are various mechanisms by which inflammation can be reduced. Naturally occurring OSCs are recognized for their anti-inflammatory and medicinal purposes, as well as for inducing diverse health benefits. On the contrary, some well-controlled studies have displayed no preventative effects of applying various forms of OSC (124, 125). Therefore, it is mandatory to ensure our understanding and examination of the efficacy and the mechanisms of action of such compounds. Oxidative stress can lead to tissue injury and inflammation by releasing inflammatory cytokines and mediators, which can also act as secondary messengers to induce the functions of NF-κB (126). Moreover, the active functional subunits of NF-κB are both p50 and p65, and the inactivation of NF-κB is carried out by binding SFN to the thiol groups of the subunits. Therefore, the presence of OSC in the diet can directly or indirectly down-regulate the activity of NF-κB to mitigate inflammatory markers (127). In this regard, adding OSC to our diet may also help reduce the pathological expression of these biomarkers, thereby providing protective support against chronic diseases (25). Despite the inverse relationship between OSC and inflammation, some studies reported opposing biological effects. For example, a concentrated dose of garlic powder (200 mg/mL) has been reported to cause significant injury in the liver (128). Moreover, the beneficial effect of garlic may be lost after chronic administration of concentrated dose (2,000 mg/kg), and this could further cause significant mortality due to myocardial injury (129). It is important to consider the optimal supplementation regime for OSC, to induce the desired effects. Concurrently, knowing the bioavailability of the selected OSC is crucial, based on how it is absorbed, metabolized and utilized by the body.

## Concluding Remark

It can be summarized that inflammation is associated with chronic disease in many ways. In addition, OSC may have an important role in preventing inflammation, as well as chronic diseases. Here, we have discussed the probable signaling pathways relating to how a reduction in inflammatory biomarkers could provide probable protection for human health against chronic diseases. Although a limited number of animal studies were evidenced, the results seem convincing, whilst *in vivo* and *in vitro* trials are being carried out to identify appropriate and feasible applications for phytonutrients, and to promote these as medicines against inflammation. Furthermore, advanced research is required to build a more thorough understanding of the anti-inflammatory functions of OSC.

## Author Contributions

RR: conceptualization, writing, and original draft preparation. KS and SM: review and editing. LR: critical evaluation and editing.

## Conflict of Interest

The authors declare that the research was conducted in the absence of any commercial or financial relationships that could be construed as a potential conflict of interest.
